# ‘You are Okay’: a support and educational program for children with mild intellectual disability and their parents with a mental illness: study protocol of a quasi-experimental design

**DOI:** 10.1186/s12888-015-0698-0

**Published:** 2015-12-24

**Authors:** Ivon Riemersma, Floor van Santvoort, Jan M. A. M. Janssens, Clemens M. H. Hosman, Karin T. M. van Doesum

**Affiliations:** Pluryn Research & Development, Industrieweg 50, 6541TW Nijmegen, The Netherlands; Behavioural Science Institute, Department Developmental Psychopathology, Radboud University, Nijmegen, The Netherlands; Professor of mental health promotion and prevention, Maastricht University and Radboud University, Nijmegen, the Netherlands Hosman Prevention Consultancy & Innovation, Groesbeek, The Netherlands; Radboud University, Nijmegen, Behavioural Science Institute/Mindfit, Mental Health Center, Deventer, The Netherlands

**Keywords:** Children, Mild ID, Parents, Mental illness, Support group, Online educational program

## Abstract

**Background:**

Children of parents with a mental illness or substance use disorder (COPMI) have an increased risk of developing social-emotional problems themselves. Fear of stigmatisation or unawareness of problems prevents children and parents from understanding each other. Little is known about COPMI with mild intellectual disabilities (ID), except that they have a high risk of developing social-emotional problems and require additional support. In this study, we introduce a program for this group, the effectiveness of which we will study using a quasi-experimental design based on matching. The program ‘You are okay’ consists of a support group for children and an online educational program for parents. The goal of the program is to increase children and parents’ perceived competence with an aim to prevent social-emotional problems in children.

**Methods/Design:**

Children between ten and twenty years old with mild ID (IQ between 50 and 85) and at least one of their parents with a mental illness will be included in the study. The children will receive part time treatment or residential care from an institute for children with mild ID and behavioural problems. Participants will be assigned to the intervention or the control group. The study has a quasi-experimental design. The children in the intervention group will join a support group, and their parents will be offered an online educational program. Children in the control group will receive care as usual, and their parents will have no extra offer. Assessments will be conducted at baseline, post-test, and follow up (6 months). Children, parents, and social workers will fill out the questionnaires.

**Discussion:**

The ‘You are okay’ program is expected to increase children and parents’ perceived competence, which can prevent (further) social-emotional problem development. Because the mental illness of parents can be related to the behavioural problems of their children, it is important that children and parents understand each other. When talking about the mental illness of parents becomes standard in children’s treatment, stigmatisation and the fear for stigmatisation can decrease.

**Trial registration:**

Dutch Trial Register NTR4845. Registered 9 October 2014.

**Electronic supplementary material:**

The online version of this article (doi:10.1186/s12888-015-0698-0) contains supplementary material, which is available to authorized users.

## Background

Research has shown that children of parents with a mental illness (COPMI) or substance use disorder are at risk of developing social-emotional problems themselves. In this study, COPMI refers to children of parents with a mental illness as well as children of parents with a substance use disorder. These children have a two to thirteen-fold higher risk of developing social-emotional or addiction problems compared to their peers with parents without a mental illness. Moreover, they also make more use of mental health care resources [1, 2].

The increased risk of COPMI can be explained by genetic, biological, and environmental factors surrounding children, their families, and their social network. Examples of genetic and biological factors include genetic predispositions and neurobiological processes during pregnancy [1, 2]. The important role of nurture and the environment in which a child grows up will form central risk factors in this study. For parents with a mental illness, it can be difficult to offer children enough regularity, safety, and positive attention. Parents can also have difficulties talking about their problems for a number of reasons. They can, for example, fear stigmatisation or be ashamed or unaware of having a mental illness, or they may not understand that their problems affect the behaviour of their children [3]. Other parents are afraid that their children might be placed out-of-home, and/or they do not want to burden their children with their problems. As a result, children and parents often do not understand each other. This can lead to these children developing feelings of guilt, shame, loneliness, and social isolation. Moreover, COPMI often show low levels of self-esteem and social acceptance [4–6].

Different preventive interventions, such as support groups, were developed for COPMI with average intelligence aimed at decreasing risk factors and improving protective factors. COPMI with mild ID are a high-risk group that has not been reached yet by preventive interventions [2]. Although little research has been conducted on COPMI with mild ID, four important risk and protective factors in both children and their parents may influence (further) social-emotional problem development. Most important risk factors are the presence of mild ID and behavioural problems in these children and the presence of a mental illness and mild ID in parents [7–9]. The combination of multiple risk factors is associated with higher risks of social-emotional problem development [2]. On the other hand, several important protective factors may prevent (further) social-emotional problem development. First, intrapersonal characteristics, such as perceived competence in both children and parents, serve as protective factors. Second, parent-child interactions, like positive parenting behaviour and parental involvement with their child’s treatment, are additional protective factors. Third, social support from children and parent’s social network can positively influence the prevention of social-emotional problems [2, 10].

Research showed that 66 % of children with mild ID and behavioural problems have a parent with a mental illness [7]. Despite these children’s increased risk and high prevalence, little research has been done on this specific group and no preventive interventions have been developed for them yet. More research is needed to test whether support groups developed for COPMI with average intelligence are effective in children with mild ID and their parents with a mental illness. COPMI with mild ID have particular needs, as described in Additional file 1 on which the ‘You are okay’-program will respond.

Over the years, various types of interventions for COPMI with average intelligence have been developed. The support group is the most common form, which is developed for children of different age groups. In these support groups, peers who have to cope with the same difficult situations come together to share experiences and feelings about coping with their parent with a mental illness. They get psycho-education to learn about their parents’ mental illness and to better understand their parent. In a safe context (small groups), they provide each other with support and practice new coping skills through, for example, role-playing games. Through stories of other children, they can learn that they are not the only ones with a parent with a mental illness and that the problems of the parent are not their fault.

The goal of these support groups is to increase protective factors and decrease risk factors. The aim is to empower the social network, take unrealistic thoughts away, increase perceived competence and strengthen the relationship between parent and child [10]. Research shows that these factors prevent (further) development of emotional and behavioural problems in COPMI and that it is possible to strengthen these factors with interventions [1, 2]. A recent RCT study has shown that support groups for COPMI aged 8 to 12 years are effective [11]. Children who participated in an intervention group sought more social support, had less unrealistic thoughts, and showed more perceived competence compared to children in the control group. Besides, these children perceived the support group as very useful, since they could share their experiences with peers.

A recommendation from the RCT study about support groups for COPMI was to further improve the involvement of parents [12]. Earlier studies showed that parental involvement is one of the most important factors in making a program for children effective [13, 14].

In addition to the interventions for COPMI, interventions for parents with a mental illness, such as an online educational program for parents with a mental illness having children with average intelligence, are being developed. It might be difficult for parents with a mental illness to join ‘live’ support groups for parents for several reasons, such as shame, low level of energy, or transport difficulties. Hence, an online educational program seems to be a more accessible way to provide parents with a mental illness parenting support. Van der Zanden et al. showed in their study with a pre-post-test design that after participating in an online educational program, parenting competence increased and emotional and behavioural problems in children decreased in comparison with baseline level [15, 16].

This study aims to examine the effectiveness of an adapted program for COPMI with mild ID. The ‘You are okay’ program consists of a support group for children with mild ID and an online educational program for their parents with a mental illness. The support group for children is based on the existing evidence-based support group for COPMI with average intelligence, and it has been adapted to the particular needs of children with mild ID [11]. Important factors that need to be considered in the adaptation of the evidence-based support group include simplifying language, providing additional sessions on basic emotions, and using examples reflecting the children’s perceptions. The adaptations are made in collaboration with professionals who work with children with mild ID and their parents. In addition, interviews with children with mild ID have provided valuable information regarding the adaptation process. Children with mild ID benefit from repeated information, especially simple and direct verbal information supported with visual information, and because of their concrete level of thinking, examples from their daily lives should be present [17]. We tested the adapted program for children with mild ID in a pilot study, the results of which have contributed to the current program and showed that children with mild ID are able to understand and reflect on their parents’ mental illness.

Parallel to the support group for children, parents will be offered an online educational program, following the recommendations of Van Santvoort on involving parents [12]. The existing online educational program for parents has been adapted for parents with children having mild ID. Simplification of language and adaptation of examples and assignments (for example if children are in residential care) were needed when adapting the parents’ online educational program because many parents of children with mild ID have mild ID themselves. They also benefit from simple use of language and concrete situations in their daily lives.

**Fig. 1:**
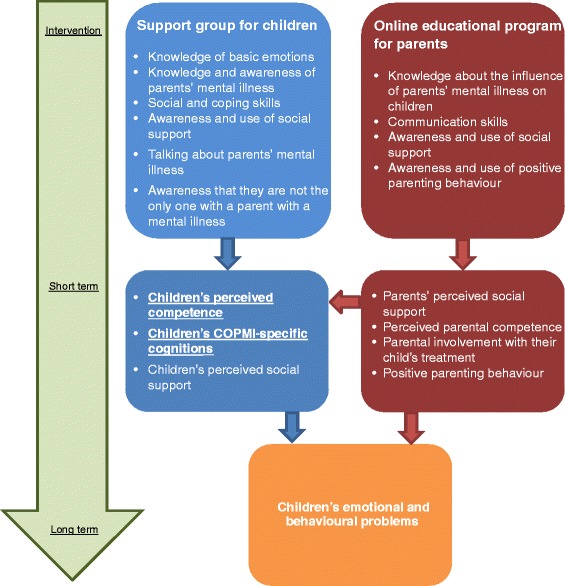
The theoretical model. This figure explains the theoretical model underlying the specialised program of ‘You are okay’

In Additional file 2, the two different parts of the ‘You are okay’ program are described. Figure 1 shows the theoretical model underlying the program based on a cognitive behavioural model. The support group for children is expected to decrease risk factors and increase protective factors in children while the online educational program for parents is expected to decrease risk factors and increase protective factors in parents. The long-term expectation is that the support group for children will contribute to less emotional and behavioural problems in children while the online educational program for parents will have a direct effect on parents and an indirect effect on children.

The aim of this study is to examine the effectiveness of the ‘You are okay’ program in increasing protective factors and decreasing risk factors in children and parents. The primary outcome measure is children’s perceived competence, which consists of COPMI-specific cognitions, self-esteem and global self-worth. Secondary outcome-measures are children and parents’ perceived social support, perceived parental competence, parental involvement with their child’s treatment, positive parenting behaviour, and children’s emotional and behavioural problems.

## Methods/Design

### Design

**Fig. 2:**
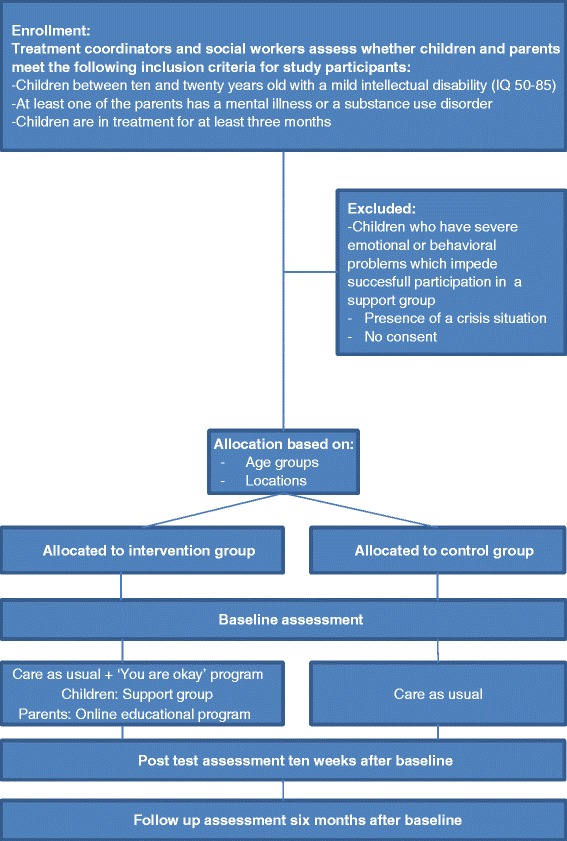
Study design. This figure illustrates the study design with the allocation, inclusion and exclusion criteria, and different assessments

This study involves a quasi-experimental design with two conditions: an intervention group and a control group. Figure 2 shows the study design with the inclusion and exclusion criteria and the group allocation and the assessments at baseline, post-test and follow up.

### Participants

Participants will be 10–20 year old children being treated for mild ID and behavioural problems at specialised institutes in the Netherlands and their parents with a mental illness. This age group is chosen because it corresponds with the age of the children in these institutes. The participating institutes specialise in treating children with mild ID and behavioural problems and offer children both residential care and part time treatment, depending on the severity of the problems of the children and their families. The researchers asked the institutes to participate in this study because of their population of children with mild ID.

The selected children had to have reached some stability in their lives in order to be able to reflect on their parent’s mental illness. Hence, the children who have been treated for at least three months, have not been in a crisis, and have been stable enough to handle potential emotional responses to the program, as indicated by the treatment coordinator, will be included in the study. Children who are not able to participate in a support group because of, for example, severe emotional or behavioural problems, will be excluded. In addition, children will be excluded if parents do not give consent, e.g., parents disagree with the child discussing the parent’s illness with others.

Many different support groups for different age groups of COPMI with average intelligence have been developed all based on the same principles. In the program ‘You are okay’, the experimental groups will formed according to children’s age; thus, age-groups will be formed. In the support group manual, different methods of the same exercises are described to match the needs of the specific age groups.

Treatment coordinators and social workers will select children whose parents acknowledge that they have a diagnosed mental illness. If there is no official diagnosis of a mental illness, but it can be assumed based on the contact with the parent and the parent’s acknowledgement of a mental illness, the parents may also be included. The severity of the parental mental health problems will be measured at baseline. If children have little or no contact with their parents, their family guardian has to agree with their participation and acknowledge that one of the parents has a mental illness. Both parents will be invited to participate in the study to complete the assessments. If the child has no contact with one of the parents, only the involved parent will be invited to participate, even if this is the parent without a mental illness. The parent who will complete the assessments will also be invited to join the educational program for parents. In addition to biological parents, other caregivers can participate, for example, foster parents or stepparents. When children perceive these persons as important caregivers, they can fulfil similar roles as biological parents.

### Procedure

Treatment coordinators and social workers will screen children and parents to be included in the study. The aims of the study will be explained to parents by phone or during a face-to-face appointment. Children and parents (or family guardians when involved) who will agree to participate and sign the informed consent form will be included. After inclusion, allocation will take place. Interviewers will administer baseline assessment to children and parents.

Assessments in both intervention and control group will be administered orally with a standardized protocol. Participants will be assessed at baseline, post-test assessment and follow up. Interviewers will visit and administer the questionnaires to children. Parents will be visited by interviewers or will be asked to answer the questions by phone. Because both children and parents might show difficulties understanding written information, simple language will be used in the information letters. Assistance will be provided to help participants complete the forms, and the study will be explained to children and parents face-to-face, allowing participants to ask their questions directly. At the same time, the interviewer will be able to check whether they understand the information. Because of the individual assessment appointments, a low attrition rate is expected. Participants will receive a reward of 5 euros after completing all assessments.

The Medical Research Ethics Committee (Independent Review Board Nijmegen) approved the study’s protocol (NL49448.072.14). The study protocol is registered at the Dutch Trial Register (NTR4845).

### Allocation

Using a quasi-experimental design, each child and his or her parent will be assigned to a condition (experimental group or control group) based on matching. Because in practice it is not possible and appropriate to form support groups with children of different ages and from different locations, matching based on age, gender, type of setting (residential or part-time) and location will be used. Therefore, it will be possible to start support groups in different locations with children of different age groups and an equal distribution of both genders and type of treatment. An independent researcher will perform the allocation before baseline assessment takes place and will assure that the experimental and control group will match.

### Sample size

Based on the existing research on the effectiveness of psychosocial interventions in children with mild ID and behavioural problems, the expectation is to find a small to medium effect on perceived competence [18]. A power analysis (two-tailed, alpha .05, statistic power 0.80) with two groups showed that 54 participants are necessary to show a small to medium effect. This calculation is based on a univariate analysis of variance with a baseline assessment, an assessment after the program, and a follow-up assessment with two groups. To explore the interaction-effects, four groups are needed, as both groups will be split again (e.g., boys versus girls) into both control group and experimental group. A power analysis (two-tailed, alpha .05, statistic power 0.80) with four groups showed that 80 participants are necessary to show a small to medium effect [19]. To compensate for 10 % attrition, the sample size needs to be 90 participants (children and parents) at minimum.

### Intervention group

The intervention group will receive the program ‘You are okay’, in addition to care as usual. The program includes a support group for children and an online educational program for parents. The support group will consist of ten weekly sessions of 1.5 h with a booster session after six weeks. Each session will have the same structure but different themes. Each session will start with a warming up exercise. After this introduction, previous week’s experiences and homework assignments will be discussed. The program will continue with exercises, including role playing games, psycho-education, videos and practising coping and social skills. At the end of each session, the session will be summarized and the homework assessment will be explained. The themes of the sessions are shown in Additional file 2, and they are linked to the theoretical model explained in Fig. 1. Two therapists who work with children with mild ID will lead the support group. The therapists will be trained to ensure that they are familiar with the program materials and that they understand the theoretical model underlying the support group. The therapists will evaluate the program and each session individually. They will also exchange their experiences during intervision meetings.

In parallel to the support group for children, parents will be offered an online educational program. Parents will receive information on five themes, reflect on their role as a parent, and learn about the possible influence of their problems on their child. The five themes are shown in Additional file 2, and they are linked to the theoretical model described in Fig. 1. Parents will complete the educational program online at home. If necessary, a social worker who is involved in the family system, will visit them three times at home to complete the educational program. The social workers are trained in using the online educational program to support parents.

### Care as usual

Children in the control group will receive care as usual from the specialised institutes for children with mild ID and behavioural problems. These institutes formulate personal treatment goals for children in a so called “individual treatment program” (for instance, a goal could be to improve the interaction with peers or to learn to cope with feelings of anger without using verbal or physical aggression). A multidisciplinary team is always involved in the treatment program of each child.

### Assessments

Instruments will be adjusted to help children with mild ID complete the assessments, with the use of colours and emoticons corresponding the response options. According to the pilot study, the assessments are appropriate for children with mild ID.

The primary outcome will be children’s perceived competence, which consists of global self-worth, social acceptance, and COPMI-specific cognitions. It will be measured using the Dutch version of the Self Perception Profile for Adolescents (SPPA) [20, 21]. Children will complete two subscales: global self-worth and social acceptance. Each subscale contains five items measured on a four-point Likert scales. Different colours will be used to underline the response options. The reliability of this instrument is sufficient [20]. This instrument has been widely used by researchers in the field of intellectual disabilities [22].

Children’s perceived competence will also be measured using a short questionnaire on COPMI-specific cognitions developed by Van Santvoort [11]. The scale assesses whether a child experiences guilt, shame, and loneliness in relation to his/her parent with a mental illness. It contains three items measured on a five-point Likert scale, which together measure the COPMI-specific cognitions. The written response options are accompanied by emoticons. According to the study by Van Santvoort [11], these three items were moderate to highly correlated with each other.

Secondary outcomes will be children and parents’ perceived social support, perceived parental competence, parental involvement with their child’s treatment, positive parenting behaviour and children’s emotional and behavioural problems.

Children and parents’ social support will be assessed using a Dutch version of the Network of Relationships Inventory – Behavioural System Version (NRI-BSV) [23]. Children and parents will complete three of the eight subscales: ‘seeks safe haven’, ‘seeks secure base’ and ‘companionship’. Each subscale originally contained three questions measured on a five-point Likert scale. In the version designed for children, the five-point Likert scale was changed to a three-point Likert scale supported by colours, following the results from the pilot study. The reliability of this instrument was proven to be sufficient [23]. Parents’ social support will also be measured using the NRI-BSV and a short version of the Dutch questionnaire Social support list-interactions (SSL-12-I) [24]. It contains 12 items measured on a four-point Likert scale, and it measures the level of social support using three subscales: everyday support, support in problem situations and esteem support. The NRI-BSV is proven to be a valid instrument [24].

Two subscales, a competence scale and an incompetence scale, taken from different questionnaires will measure parental competence. The competence will be measured using the Parenting Self-Agency Measures [25]. It contains five items measured on a six-point Likert scale and assesses the perceived efficiency as a parent. The incompetence scale will be measured using a short version of the Dutch questionnaire ‘Nijmeegse ouderlijke stress index (Nijmegen parental stress index)’ [26]. It contains six items measured on a six-point Likert scale and assesses feelings of inadequacy as a parent. Both subscales have sufficient reliabilities [26, 27].

Parental involvement with their child’s treatment will be measured using a single question measured with a scale from 0 to 10 developed for the purpose of this study. It measures the degree of involvement of the parents in the treatment of the child.

Positive parenting behaviour will be measured using the Parenting Scale [27], which has been tested and appears to be a valid and reliable instrument. Parents will complete subscales laxness and over reactivity measured by 21 items on a seven-point Likert scale.

Children’s emotional and behavioural problems will be assessed using the Strengths and Difficulties Questionnaire (SDQ) [28, 29]. Children, parents and social workers will be asked to complete this 25-item questionnaire measured on three-point Likert scales. The questionnaire for children will include colour coded response options. This instrument assesses the strengths and difficulties of children in five domains: emotional symptoms, conduct problems, hyperactivity-inattention, peer problems and prosocial behaviour. Its reliability and validity is well established [30].

The following questionnaires will be used to study interaction effects. The severity of the parents mental illnesses will be measured using the Brief Symptom Inventory (BSI) [31] and parents’ well-being will be measured using the WHO-Five Well-being Index (WHO-5) [32]. The treatment coordinators of the children will provide background information to study other interaction effects (gender, age, country of birth, IQ, diagnoses of the child, type of treatment).

### Statistical analysis

The data from all participants in experimental and control groups will be analysed according to the intent-to-treat principle. Missing data will be estimated with multiple imputation. The effectiveness of the program will be analysed with a multi-level path analysis in Mplus to account for differences between and within locations. The experimental and control group will be compared at baseline, post-test and follow up. We will control for baseline differences between the experimental and control group in demographic variables (e.g., gender and cultural background). Interaction effects will be analysed, as various studies on other interventions reported that the effectiveness of these interventions varies with differences in these variables [33–35]. All interaction effects will be studied in an explorative way with 2x2 ANOVA’s, with the experimental and control group each comprising two levels.

## Discussion

Little research has been conducted on COPMI with mild ID and preventive interventions to prevent (further) development of social-emotional problems. What we do know is that this population has an increased risk of developing social-emotional problems and that many children with mild ID in residential care settings or in part-time treatment also have a parent with a mental illness.

Until now, the treatment for children with mild ID and behavioural problems has focused mainly on the problems and goals of the children while overlooking the mental health problems of parents. Although several preventive interventions have been developed for COPMI with average intelligence, no intervention programs have been offered to this vulnerable group. The parental awareness of the influence of their mental illness on children is insufficient. In addition to the child-centred focus of treatment facilities, stigmatisation problems may prevent parents from talking about their mental illness both with their children and with professionals. Consequently, children may not be able to understand why they cannot live with their parents. The involvement of parents with their child’s treatment is an important factor in improving this understanding. Children need to understand the behaviour of their parent in relation to their mental illness and parents need to understand the influence of their mental illness on the behaviour of their child. Although the involvement of parents is important, it is not always easy to engage parents. Many parents are inclined to refuse help due to their negative experiences with treatment facilities and social workers. Hence, it is often challenging to involve them in their child’s treatment.

Children often demonstrate feelings of loneliness because of the lack of social support from their parent or because they think that they are the only ones with a parent with a mental illness. We expect that these COPMI specific cognitions of loneliness, shame and guilt will decrease when children take part in specialised support groups and their parents join an online educational program. In the long term, we expect that emotional and behavioural problems in children will decrease and (further) development of mental health problems will be prevented.

The aim of this study will be to test the effectiveness of the program ‘You are okay’ for children with mild ID and their parents with a mental illness or substance use disorder. Once effectiveness is demonstrated, the program would be eligible for regular implementation in practice.

### Strengths and limitations

One of the strengths of this study is that the program will be based on an existing evidence-based support group [11]. The core principles of this evidence-based intervention will remain intact in the program ‘You are okay’. Second, adaptions are made to fit the needs of children with mild ID. The results of the Van Santvoorts’ study [11] have been used to improve the content of the support group, e.g., the involvement of parents was found to be an important factor. In addition to the children’s support group, the program ‘You are okay’ consists of an online educational program for parents to improve their involvement. Third, the adapted program has already been tested in a pilot study. The program and assessments were checked in terms of whether they matched the needs of children with mild ID and their parents and some adaptations were made based on these experiences. Finally, in order to prevent high rates of attrition, we will inform parents and children personally about the study, and we will also help them complete the questionnaires.

A limitation of this study is that it might be difficult to enrol the participants due to the fear of stigmatisation, as it might be difficult for parents to talk about their problems. They might have difficulties acknowledging their mental illness and admitting to children and professionals that they indeed have problems. Furthermore, it might be difficult to obtain permission from parents for their child to participate in the study. Second, many parents do not have an official diagnosis of a mental illness because the participating institutes focus primarily on the child’s treatment. Informants, like social workers who are involved with these families, provide the necessary information.

### Implications for practice

If the treatment facilities implement the’You are okay’-program, it may also help to reduce the fear of stigmatisation in order to get parents more closely involved in their child’s treatment. Treatment facilities should broaden their focus from the current, mainly child-centred support to family-centred. It is important to consider the important role and influence of the entire family system and child’s environment, although this would require much effort from professionals. For example, children do not live in residential care settings only because of the severity of their ID and additional behavioural problems. Often, out-of-home placement is the result of a combination of their needs and parents who are not able to fulfil these particular needs, for example, because of their own mental illness. These complex situations are often not communicated among children, parents, and social workers. We expect that if the silence around the parent’s mental illnesses is broken and interaction between children and their parents improves, children will be better able to grasp the situation and understand their parent. When openness and understanding each other will be stimulated, both children and parents will probably feel increased competence and a higher quality of life.

## Additional files

Additional file 1:
**COPMI with mild ID and their parents.** This file contains information about children with mild ID and their parents, which is illustrated by a case from one of the participating health care facilities. (DOC 27 kb) Additional file 2:
**The ‘You are okay’-program.** Description: This file contains detailed information about the specialised developed program, ‘You are okay,’ including the methods and themes. (DOC 24 kb)

## Abbreviations

COPMI: Children of parents with a mental illness
ID: Intellectual disability
