# Supplementary material for: ‘You are Okay’: a support and educational program for children with mild intellectual disability and their parents with a mental illness: study protocol of a quasi-experimental design
Source: BMC Psychiatry. 2015 Dec 24;15:318. doi: 10.1186/s12888-015-0698-0 (PMC4690258; doi:10.1186/s12888-015-0698-0)
Supplement: Additional file 1: — COPMI with mild ID and their parents. This file contains information about children with mild ID and their parents, which is illustrated by a case from one of the participating health care facilities. (DOC 27 kb) [file 12888_2015_698_MOESM1_ESM.doc]

**Additional file 1: COPMI with mild ID and their parents**

Children with mild ID have an IQ between 50 and 85, also called borderline IQ. There is a difference between the mental age and actual age of children with mild ID in that the mental age is lower than the actual age. Many children with mild ID also have diagnosed behavioural problems, for example, autism or conduct disorder [36]. Specific deficits or vulnerabilities associated with children’s mild ID can explain the high prevalence of different behavioural problems in these children. First, children with mild ID differ from children with average intelligence in social information processing; they encode more negative information, and they have less assertive and more submissive and aggressive problem solving skills [37]. Second, children with mild ID show difficulties in executive functions, such as selective attention, working memory, and inhibition [8]. Most children with mild ID therefore need specialised treatment to obtain a positive development and to prevent (further) problem development. The degree of the behavioural problems in children and the treatment intensity differ widely. Some children with mild ID live in residential care settings while others live at home with their parents and receive part time treatment from an institute for children with mild ID and behavioural problems.

Parenting children with mild ID and behavioural problems can be challenging for healthy parents. Parents of children with mild ID report more stress compared to parents from children with average intelligence and if children also have behavioural problems, parental stress increases even more [10]. If parents have a mental illness (or mild ID) themselves, parenting can be extra difficult, due to the stress from their own problems. Many parents of children with mild ID have a mental illness [10]. Often no balance exists between the needs of the children and the capacity of the parents, which results in treatment in a (residential) care setting.

*Case: Peter*

*Peter is a 10-year old boy with mild ID. He lives in a residential care setting for children with mild ID and behavioural problems. Peter receives treatment to learn how to cope with feelings of anger, without using verbal or physical aggression. Peter asks group workers frequently why he cannot go home and why he has to live in the residential care setting. He thinks that it his fault he lives here and that he cannot go home. He has low self-esteem. Peter’s parents are not able to take care of Peter, and they are supported by a family guardian. His mother has a substance use disorder. Peter’s parents do not talk with Peter about his mother’s problems. They think that he is not aware of these problems and that he is too young to be told.*
