# Supplementary material for: ‘You are Okay’: a support and educational program for children with mild intellectual disability and their parents with a mental illness: study protocol of a quasi-experimental design
Source: BMC Psychiatry. 2015 Dec 24;15:318. doi: 10.1186/s12888-015-0698-0 (PMC4690258; doi:10.1186/s12888-015-0698-0)
Supplement: Additional file 2: — The ‘You are okay’-program. Description: This file contains detailed information about the specialised developed program, ‘You are okay,’ including the methods and themes. (DOC 24 kb) [file 12888_2015_698_MOESM2_ESM.doc]

**Additional file 2: The ‘You are okay’ program**

Support group for children with mild ID and parents with a mental illness

- Ten weekly sessions and a booster session after six weeks
- Social support from peers with comparable home situations
- Methods: Psycho-education, training social and coping skills, social support
- Themes: get to know each other, recognize problems at home, recognize basic emotions, show emotions, understand mental illnesses, use social network, cope with difficult situations, develop social skills and parting session

Online educational program for parents with a mental illness and children with mild ID

- Online information with homework assignments and videos
- When necessary with assistance in three sessions
- Methods: Psycho-education
- Themes: negative cognitions, possible influence of problems on the children, communication, positive behaviour of the children and social network
